# Evaluation of high-pressure homogenization modified quinoa protein on water distribution, gel and rheology properties of chicken breast batters

**DOI:** 10.1016/j.fochx.2026.104353

**Published:** 2026-08-21

**Authors:** Yanyan Zhao, Xinyuan Yao, Qizhao Han, Zhaohui Wei, Ligong Zhai, Shengming Zhao, Guoyuan Xiong

**Affiliations:** a School of Food Science and Engineering, Anhui Science and Technology University, No. 999 Wenhui Road, Chuzhou 239000, PR China; b School of Food Science and Technology, Henan Institute of Science and Technology, No. 90 Hua lan Street, Xinxiang 453003, PR China; c Anhui Province Key Laboratory of Functional Agriculture and Functional Food, Anhui Science and Technology University, Chuzhou 239000, PR China

**Keywords:** High pressure homogenization modified quinoa protein, Chicken breast batters, Gel properties, Rheological properties, Water distribution

## Abstract

This study examined the influence of quinoa protein modified by high-pressure homogenization (HQP) on the gelation behavior, water mobility, rheological attributes, and microstructure of chicken breast batters. Cooking yield and gel strength of batter gels increased progressively from 88.93% and 859.56 g to 95.35% and 1467.34 g, respectively, with elevated HQP supplements. Concurrently, *L*^⁎^ and *a*^⁎^ values dropped gradually, whereas the *b*^⁎^ value elevated evidently (*P* < 0.05). Textural indicators all displayed an ascending-descending trend, reaching optimal values at 12% HQP incorporation. Rheological assessment demonstrated that HQP markedly enhanced the G' value throughout thermal and frequency scan analyses. Low-field nuclear magnetic resonance (LF-NMR) data indicated that HQP significantly elevated the immobilized water fraction while concomitantly reduced the free water proportion. Fourier transform infrared (FTIR) spectroscopy indicated a marked decline in α-helix fraction paired with elevated β-sheet formation. SEM imaging confirmed that HQP supplementation facilitated the formation of a smoother and more compact gel architecture. Collectively, HQP demonstrates strong potential as a functional ingredient for enhancing emulsified chicken systems.

## 1 Introduction

Meat batters have consistently enjoyed widespread consumer acceptance owing to their distinctive flavor, elastic texture, high nutritional value, and convenience. As a complex multiphase colloidal system, meat batter comprises water, proteins, lipids, and soluble non-protein constituents (Ghafouri-Oskuei et al., 2022). Its gelation capacity is intimately associated with critical quality attributes, including hardness, adhesiveness, springiness, water retention, and cooking yield (Liu, Wang, et al., 2023; Liu, Yang, et al., 2023). These gel characteristics are fundamentally governed by the 3D network architecture generated via thermal aggregation and cross-linking of myofibrillar proteins (Hou et al., 2025). Enhanced protein cross-linking generates a denser matrix, thereby improving texture and water retention in the final products. However, relative to pork batters, chicken batters exhibit inferior gel properties, water-holding capacity, and textural attributes upon thermal processing, falling short of consumer expectations for premium comminuted meat emulsions (Zheng et al., 2021). Protein concentration represents a principal determinant of the physicochemical and functional attributes of meat batter systems, with elevated protein levels promoting gel matrix development and enhancing overall product quality. Consequently, the incorporation of non-meat proteins to ameliorate gel quality has emerged as an effective strategy, as substantiated by recent investigations. Ma, Sang, Wang, Zhang, and Guo (2025) demonstrated that HPH-modified chickpea protein supplementation significantly reduced the cooking loss while improved water-holding capacity and gel strength in chicken batters. Similarly, Xie, Zou, Li, Kang, and Ma (2022) reported that the addition of high pressure treated soy protein effectively enhanced the texture of pork batter.

Quinoa (*Chenopodium quinoa*), one of the few edible species within the Chenopodiaceae family, has witnessed remarkable production growth in China, with annual output reaching approximately 300,000–500,000 metric tons in 2025, ranking the country third globally. With a protein content reaching 15% (dry weight basis), quinoa substantially outperforms conventional cereals in protein density. Its protein quality, underpinned by the presence of all nine indispensable amino acids in proportions akin to bovine casein, confers recognition as a nutritionally adequate complete protein (Zhao et al., 2023). Quinoa protein is predominantly composed of albumin (35%, 50–75 kDa) and globulin (37%, 80–90 kDa). Quinoa protein emerged as a premium ingredient for health food development over the past decade, attributed to its exceptional nutritional composition and hypoallergenic nature (Wang, Miao, & Sun, 2024). Cen et al. (2022) reported that 5.0%–7.5% quinoa protein addition significantly enhanced the gel strength of threadfin bream myofibrillar protein gels. Similarly, Zhao et al. (2024) established that quinoa protein effectively enhanced the gel properties of carp surimi. Nevertheless, the poor solubility of native quinoa protein has constrained its broader application in food processing. Modification of protein structure is pivotal for improving functional properties, and physical field treatments have attracted considerable attention as green, safe, and chemical-free alternatives. While ultrasound, pulsed electric field (PEF), and high-pressure homogenization (HPH) have all been employed for protein modification, they differ markedly in their limitations. Ultrasound introduces localized heating and free radical-mediated oxidation (Ampofo & Ngadi, 2022), and PEF remains constrained by high equipment costs and limited scalability (Ekezie, Cheng, & Sun, 2018). By contrast, HPH leverages mechanical shear, cavitation, and impingement to achieve controlled structural rearrangement under ambient conditions, without the thermal or oxidative liabilities of other methods. Its advantages extend further to homogeneous treatment, straightforward scale-up, and inherent suitability for heat-sensitive proteins, which collectively underscore its superiority for industrial protein functionalization (Zhao et al., 2023). Our previous research revealed that HPH modification effectively ameliorates the physicochemical and functional attributes of quinoa protein through intense mechanical forces (shear and cavitation), which induce controlled protein denaturation, reduce particle size, modify surface characteristics, and disrupt tertiary structures (Zhao et al., 2023). Related studies have further demonstrated that HPH modified plant proteins, such as soybean protein significantly improve the gelation characteristics of pork myofibrillar protein (Ge et al., 2023). However, quinoa protein distinguishes itself from these conventional plant proteins by several inherent advantages. As a rare complete plant protein, quinoa protein contains all nine essential amino acids with a well-balanced profile, particularly exhibiting higher levels of lysine and methionine compared to most cereal and legume proteins. Furthermore, unlike soy protein, quinoa protein is free from major plant allergens (gluten, soy, and nut allergens), rendering it a safer and more inclusive ingredient for consumers with food sensitivities (Dakhili, Abdolalizadeh, Hosseini, Shojaee-Aliabadi, & Mirmoghtadaie, 2019). Despite these distinctive merits, as far as we are aware, no existing investigation has elucidated the influence of HPH-modified quinoa protein (HQP) on chicken breast batter gelation.

Accordingly, the present work was designed to systematically elucidate the effect of HQP addition at varying concentrations on cooking yield, color attributes, textural properties, gel strength, rheological behavior, water mobility, protein secondary structure, and microstructural characteristics of chicken breast batter gels. The aim was to elucidate the modulatory effects of HQP on chicken batter gelation, thereby establishing a technical basis for the utilization of HQP in premium meat product innovation.

## 2 Materials and methods

### 2.1 Materials

Chilled chicken breast (six-week-old white-feathered chicken, 2.5 ± 0.2 kg in weight, female, 24 h post-mortem, pH 5.92), derived from a single batch of broilers, was collected from a municipal market in Chuzhou, China. Prior to analysis, visible adipose tissue, vascular structures, and superficial fascia were manually excised. The samples were portioned into uniform cubes (approximately 0.5 cm^3^), and frozen at −20 °C for up to 14 days prior to further evaluation. Dry quinoa was provided by an Ecological Agriculture Co., Ltd. (Zaozhuang, China).

### 2.2 Quinoa protein extraction and HQP preparation

Dry quinoa was ground to 40-mesh. Protein was extracted by ultrasonic-assisted alkali: powder dispersed in water (1,35), pH adjusted to 10 with 1 mol/L NaOH, ultrasonicated (400 W, 40 °C, 120 min), centrifuged (8000×*g*, 10 min, 4 °C), precipitated the supernatant at pH 4.5 (30 min), adjusted to pH 7.0, freeze-dried, and stored. Freeze-dried quinoa protein was reconstituted in ultrapure water (10 mg/mL), agitated for 1 h, and homogenized (FGP 12805, Stansted Fluid Power Ltd., UK) at 120 MPa for 3 cycles. The obtained HQP suspensions were vacuum freeze-dried and stored for later analyses (Zhao et al., 2023).

### 2.3 Preparation of chicken breast batter gels

Following refrigerated thawing (4 °C, 12 h), 200 g of ground chicken breast was combined with 40 mL of ice-cold water, 2% (w/w) sodium chloride, and various mass proportions of HQP (0%, 3%, 6%, 9%, 12%, and 15%). The mixture was chopped for 2 min using a vacuum chopper (Stephan UMC-5C, SMG Co., Hameln, Germany), and this operation was repeated twice, followed by a final 2 min chopping cycle to obtain HQP-chicken breast batters. During processing, the final temperature was maintained below 10 °C. Portions (40 g) of the batter were transferred to polypropylene vacuum pouches and molded into cylinders. The samples were thermally treated (85 °C, 30 min), cooled, and held at 4 °C for 12 h prior to subsequent testing (Ma et al., 2025).

### 2.4 Cooking yield

Following the procedure of Lv et al. (2021), the chicken breast batters were weighed prior to heating (designated as W₁) and were subsequently subjected to a heat treatment at 85 °C for 30 min. After cooling for 2 h, surface moisture was removed with filter paper before the sample was reweighed (W₂). The cooking yield was computed using the following mathematical expression:

(1) $$\mathrm{Cooking yield}\;\left(\%\right)=\frac{W₂}{W₁}\times 100\%$$

### 2.5 Color

Color differences were monitored using a CR-400 colorimeter (Konica Minolta, Japan) with a white tile as the standard reference (Zhao et al., 2024). Following refrigeration, samples were equilibrate to room temperature, cut into cylindrical specimens (15 mm diameter × 20 mm height), and six equidistant points on the cut surface were selected for color measurement. *L*^⁎^*, a*^⁎^*,* and *b*^⁎^ values were recorded to represent lightness, redness, and yellowness. The whiteness value was quantified as follows:

(2) $$\mathrm{Whiteness}=100-\frac{{\left[{\left(100-L^{*}\right)}^{2}+a^{*2}+b^{*2}\right]}^{1}}{2}$$

### 2.6 Texture and gel strength

Chicken breast batter gels were stabilized under ambient conditions for 2 h after refrigeration, then sectioned into cylindrical specimens (1.5 cm × 2 cm). Texture measurements were carried out using a textureometer (TA-XT plus Texture analyzer, Stable Micro Systems, UK) equipped with a P/36R probe in TPA mode to determine hardness, springiness, cohesiveness, and chewiness. Experiments were conducted using a 50% compression ratio, a constant speed of 2 mm/s (pre-test, mid-test, and post-test), a 5 g trigger force, and a 5 s interval. Gel strength was determined with the identical instrument in Return-To-Start mode using a P/0.5 probe. Experiments were performed with a penetration ratio of 50%, pre-test and mid-test speeds of 1 mm/s, a post-test speed of 5 mm/s, and a trigger force of 5 g (Liu, Wang, et al., 2023; Liu, Yang, et al., 2023).

### 2.7 Dynamic rheology

The rheological analysis was performed on a rotational rheometer (HAAKE MARS, Thermo Scientific, Karlsruhe, Germany) fitted with a P35 TiL parallel plate geometry (Zhao et al., 2025). The storage modulus (G′) of meat batters (approximately 3 g) was measured during temperature ramp and frequency sweep tests. For the dynamic temperature sweep, samples were equilibrated (20 °C, 5 min) and subsequently heated from 20 to 80 °C at 2 °C/min with a constant oscillatory frequency of 0.1 Hz. For the dynamic frequency sweep, samples were subjected to a frequency ramp from 0.1 to 20 Hz at 0.05 Hz/min (constant temperature: 20 °C; strain: 1%).

### 2.8 Low-field NMR

The gels were diced into small rectangular pieces and put into NMR tubes (15 mm diameter). Water distribution was assessed using a low-field NMR instrument (PQ001, Niumag Electric Co., Shanghai, China) operating at a Larmor frequency of 22.6 MHz (proton). The spin-spin relaxation time (T₂) and related water proportion (PT_2_) were monitored by a CPMG pulse sequence with 32 scans and a scan time of 2 s. The T₂ data were deconvoluted into a multi-exponential model using the Multi-Exp Inv software and then normalized (Zhao et al., 2024).

### 2.9 FT-IR spectroscopy

Lyophilized gels were pulverized and homogenized with KBr (1100, w/w). The mixture was pelletized and tested using a Fourier infrared spectrometer (TENSOR 27, BRUKER Co., Germany). Spectra were acquired using the following optimized parameters: 64 scans, mid-infrared region of 4000–400 cm^−1^, and resolution of 4 cm^−1^. Changes in protein secondary structure were quantified with OMNIC 8.0 and PeakFit 4.12 software (Ma et al., 2025).

### 2.10 Microstructure

Gel specimens were immersed in 2.5% glutaraldehyde (pH 6.8, 24 h), progressively dehydrated through ethanol gradients (50%, 70%, 90%, and 95%, 15 min each), and concluded with triple rinsing in absolute ethanol (10 min per cycle). Subsequently, the samples were defatted with chloroform (1 h), and exchanged through tert-butanol/ethanol (1:1) and pure tert-butanol (15 min each). Following vacuum dehydration at 30 °C for 4 h, the specimens were gold-sputtered and subsequently visualized by scanning electron microscope (Quanta 200, FEI Inc., Portland, USA) with a magnification of 1000× (Wang et al., 2020).

### 2.11 Statistical analysis

All experiments were independently carried out at least three times, and each experimental condition comprised four replicate measurements. Statistical analyses were performed by SPSS 23.0, and the results were depicted as mean ± SD. One-way analysis of variance (ANOVA) followed by Duncan's test was applied for multiple comparisons (*P* < 0.05).

## 3 Results and discussion

### 3.1 Cooking yield

**Fig. 1:**
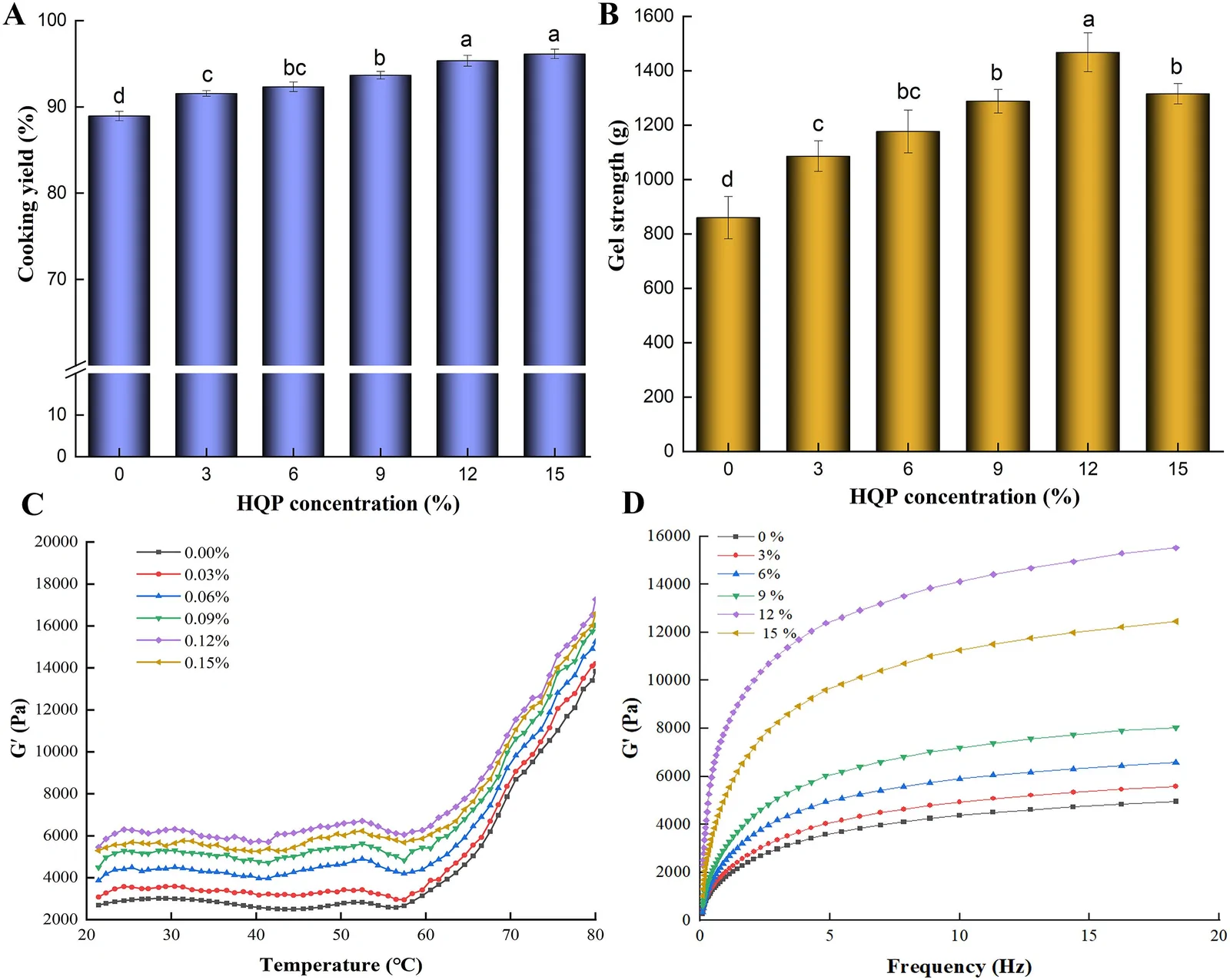
Effects of HQP on the cooking yield (A), gel strength (B), storage modulus G' in temperature scanning (C) and storage modulus G' in frequency scanning (D) of the chicken breast batter gel. Note: Different letters indicate significant differences among groups (*P* < 0.05).

Chicken breast batter gels showed a notable increase in cooking yield (Fig. 1A) with elevated HQP concentrations compared with the control (88.93%) without HQP supplementation (*P* < 0.05). At an additional level of 12%, the cooking yield reached 95.34%, representing a 6.41% increase relative to the control. This improvement is primarily ascribed to the binding of HQP to myofibrillar proteins in the chicken breast batter, which promotes additional hydrogen bond formation and enhances water retention (Cen et al., 2022). Furthermore, HPH modification unfolds the quinoa protein structure and exposes side-chain groups, thereby increasing more hydrogen bond sites, strengthening hydrogen bonding, and promoting disulfide bond formation in quinoa protein (Gul et al., 2025). During heat-induced gelation, unfolded myofibrillar proteins engage in enhanced hydrophobic interactions with HQP, facilitating the formation of a more ordered and densely packed gel network that effectively immobilizes a greater proportion of free water, thereby enhancing the water-holding capacity and cooking yield of chicken breast batter gels (Li et al., 2021). The present findings are consistent with the observations of Dong, Zhao, Wang, and Jiang (2023), who revealed that the incorporation of ultrasound-modified pea protein markedly enhanced the water-holding capacity of myofibrillar protein gels. Similarly, Lee, Jang, Kang, and Chin (2017) reported that red bean protein supplementation lowered the cooking loss of pork MP gels.

### 3.2 Color

Color serves as a pivotal determinant of consumer purchasing decisions for meat products. As illustrated in Table 1, variations in color characteristics stem principally from the inherent pigmentation of quinoa protein itself. Previous studies have shown that the color properties of composite gels are primarily governed by the intrinsic color, type, and concentration of incorporated exogenous substances (Petcharat & Benjakul, 2018). Relative to the control, progressive incorporation of HQP induced significant decreased *L*^⁎^, *a*^⁎^, and whiteness values, accompanied by a marked increase in *b*^⁎^ value. Such color changes likely result from the inherent pale yellow color of quinoa protein powder, which reduces light scattering in the meat gels, thereby decreasing *L*^⁎^, *a*^⁎^*,* and whiteness values while increasing *b*^⁎^ (Zhao et al., 2024). The decreased redness may also reflect reduced hemoglobin content in the batter, coupled with increased accumulation of oxidative pigments in muscle proteins (Niu, Xia, Wang, Kong, & Liu, 2018). This observation is consistent with the findings of Ma et al. (2025), who reported that progressive increases in HPH-modified chickpea protein concentration significantly lowered the *L*^⁎^, *a*^⁎^, and whiteness of minced chicken gels while remarkably increasing the *b*^⁎^ value (*P* < 0.05). Collectively, these chromatic alterations in HQP-enriched chicken gels imply that thermal gelation may instigate oxidative transformations in proteins and/or chromophores.

**Table 1:** Effect of HQP on the color of the chicken breast batter gel.

HQP concentration (%)	*L*^⁎^	*a*^⁎^	*b*^⁎^
0.00	86.39 ± 0.67^a^	1.62 ± 0.32^a^	12.14 ± 0.20^d^
0.03	84.24 ± 0.40^b^	1.07 ± 0.15^b^	13.18 ± 0.20^c^
0.06	83.01 ± 0.17^c^	0.98 ± 0.14^bc^	13.42 ± 0.32^c^
0.09	82.05 ± 0.31^d^	0.84 ± 0.11^c^	14.54 ± 0.26^b^
0.12	81.09 ± 0.34^e^	0.57 ± 0.03^d^	15.88 ± 0.33^a^
0.15	80.48 ± 0.10^f^	0.44 ± 0.10^d^	15.55 ± 0.36^a^

Note: Different letters in the same column indicate a significant difference (*P* < 0.05).

### 3.3 Texture and gel strength

Compared with the control, chicken breast batter gels displayed a significant rise and subsequent fall in textural parameters as the HQP addition level elevated (Table 2). The peak values of hardness, springiness, cohesiveness, and chewiness were attained at a 12% addition level, reaching 10,839.82 g, 0.91, 0.75, and 7553.54 g.mm, respectively, which corresponded to increases of 95.15%, 13.75%, 38.89%, and 121.62% relative to the control. The disproportionate increase in chewiness, which is a composite function of hardness, cohesiveness, and springiness, implies that HQP addition primarily enhanced the energy required to fracture the gel network rather than merely increasing its elastic recovery. This indicates that HQP facilitated the formation of a more stress-resistant, fracture-tolerant protein matrix rather than simply increasing cross-link density uniformly. The primary cause of this phenomenon is the excellent gelling and emulsifying properties inherent to quinoa protein (Yang et al., 2022). Furthermore, HPH modification induced the exposure of additional hydrophobic groups, facilitating interactions and cross-linking between HPQ and myofibrillar proteins within the chicken batter (Zhao et al., 2023). During thermal processing, these activated HQP molecules likely act as “molecular bridges” between myofibrillar protein aggregates. The exposed hydrophobic patches on HQP can interact with hydrophobic domains on myosin and actin that become accessible during thermal denaturation, while the increased sulfhydryl content may contribute to inter-protein disulfide cross-linking (Kang et al., 2022). As also noted by Xie et al. (2022), the ultra-high pressure modified soybean protein addition enhanced the hardness, elasticity, and chewiness of pork batters by promoting an increase in surface binding sites of soybean protein, thus promoting interactions between soybean and myofibrillar proteins. Zhang, Pan, Jiang, and Shi (2023) found similar results, demonstrating that the incorporation of soy protein isolates substantially enhanced the hardness and chewiness of low-salt surimi gel. The deterioration of textural parameters at 15% HQP addition indicates a disruption of the gel network architecture. We hypothesize that excessive HQP may compete with myofibrillar proteins for available water, effectively diluting the myofibrillar protein concentration below its critical gelling threshold. Alternatively, at high concentrations, HQP may undergo self-aggregation preferentially over co-aggregation with myofibrillar proteins, creating protein-rich domains that phase-separate from the myofibrillar network. This would introduce structural defects and stress-concentration points within the gel matrix, reducing its mechanical integrity (Zhang, Zhang, Chen, He, & Wang, 2022). This interpretation aligns with Zhao et al. (2024), who reported that excessive non-muscle protein can sterically hinder myofibrillar protein aggregation during thermal gelation.

**Table 2:** Effect of HQP on textural properties of the chicken breast batter gel.

HQP concentration (%)	Hardness (g)	Springiness	Cohesiveness	Chewiness (g.mm)
0.00	5554.64 ± 221.11^e^	0.80 ± 0.01^c^	0.54 ± 0.01^e^	3408.60 ± 176.28^e^
0.03	7262.29 ± 412.86^d^	0.83 ± 0.01b^c^	0.58 ± 0.01^d^	4524.12 ± 293.27^d^
0.06	8141.08 ± 294.71^c^	0.86 ± 0.02^b^	0.65 ± 0.03^c^	6013.70 ± 207.95^c^
0.09	9586.39 ± 453.85^b^	0.87 ± 0.01^b^	0.70 ± 0.01^b^	6861.83 ± 223.83^b^
0.12	10,839.82 ± 156.89^a^	0.91 ± 0.02^a^	0.75 ± 0.01^a^	7553.54 ± 202.26^a^
0.15	9618.84 ± 116.00^b^	0.85 ± 0.02^b^	0.72 ± 0.01^b^	7012.23 ± 220.05^b^

Note: Different letters in the same column indicate a significant difference (*P* < 0.05).

As evident from Fig. 1B, unlike the control (859.56 g), the gel strength of chicken meat batters increased significantly and then decreased with increasing HQP concentration (*P* < 0.05), peaking at 1467.36 g at a 12% addition level. Gel strength reflects the overall integrity and rupture resistance of the 3D protein network. This improvement presumably stems from the ability of HQP particles—rendered smaller and more soluble by HPH treatment—to permeate and occupy interstitial voids within the myofibrillar protein gel network, thereby effectively increasing the solid volume fraction and stress-bearing capacity (Cen et al., 2022; Zhao et al., 2022). Moreover, HPH treatment triggered the surface exposure of a greater number of hydrophobic groups, creating more binding sites for myofibrillar proteins and promoting the HQP- myofibrillar protein hydrophobic interactions throughout the thermal gelation process, thereby facilitating the establishment of a dense, well-structured protein gel network accompanied by boosted gel strength (Ge et al., 2023). This is mechanistically distinct from simple filler reinforcement; rather, it represents active network modification where HQP integrates into the gel framework instead of merely occupying space. Findings from our prior research indicate that HPH treatment of quinoa protein reduces particle size and enhances solubility, thereby facilitating the establishment of a tightly organized gel framework in chicken meat batter gels (Zhao et al., 2023). Conversely, elevating the concentration to 15% significantly decreased gel strength, which further supports the hypothesis of network disruption at excessive HQP concentrations. Beyond the optimal level, the competitive hydration and potential phase separation of HQP may create a bicontinuous or phase-inverted microstructure where the continuous myofibrillar protein phase loses its dominance. This would manifest macroscopically as reduced gel strength despite the higher total protein content (Zhao et al., 2024). The present results mirror those reported by Dong et al. (2023), who revealed that adding ultrasound-modified pea protein effectively elevated the gel strength of pork myofibrillar protein gels. Similarly, Wang et al. (2025) confirmed that addition of HPH-modified chickpea protein significantly enhanced the gel strength of reduced-phosphate myofibrillar protein gels.

### 3.4 Dynamic rheology

Dynamic rheological measurements serve as vital benchmarks for evaluating gel elasticity and structural integrity. Fig. 1C illustrates the impact of HQP concentration on the G′ value of chicken breast batters during a temperature sweep. The G′ evolution of chicken breast batters during thermal processing exhibited three distinct stages. In the first stage (20–52 °C), G′ increased slightly and then decreased gradually, corresponding to the initial gelation of the meat batter driven by protein-protein interactions. In the second stage (52–58 °C), G′ decreased sharply, potentially attributable to protein denaturation under raised thermal conditions, which disrupted the fragile gel structure formed earlier. In the third stage (58–78 °C), G′ increased rapidly, primarily due to enhanced protein interactions, re-aggregation, and cross-linking at higher temperatures, as the meat batter transformed from a viscous semi-solid into a uniform, durable 3D network architecture (Wang et al., 2025). Fig. 1C indicates that the G′ curves of both the control and treated groups displayed the classical rheological profile characteristic of chicken breast batters, indicating that heating induced protein aggregation to varying extents. With increasing HQP concentration, all treated groups showed a progressive rise in G′ values with temperature. Notably, the maximum G′ value was observed at a 12% addition level. This primarily stems from the partial structural unfolding of the quinoa protein induced by HPH, which exposed previously buried active groups and maintained the protein in a molten globule conformation, thereby accelerating interactions between the HQP and myofibrillar proteins and facilitating the assembly of a dense gel architecture (Liu, Geng, Zhao, Chen, & Kong, 2015). Moreover, the improved gel strength of chicken breast batters upon HQP (Fig. 1B) likely contributed to the elevated G′ values. HQP can function as a filler within meat protein networks, thereby enhancing the elastic properties of chicken breast batters (Cen et al., 2022). However, at an excessive addition level (15%), G′ began to decline, possibly attributable to the high solubility of the HQP, which increasing the fluidity of the system at elevated concentrations (Lv et al., 2021).

The G′ value obtained during the oscillation sweep reflects the elastic component of viscoelastic behavior, representing the recoverable energy stored per oscillation cycle. Elevated G′ values are indicative of stronger intermolecular interactions, enhanced elastic stability, and greater gel strength (Cao, Xia, Zhou, & Xu, 2012). As evidenced by Fig. 1D, the G′ values of chicken breast batters rose with escalating oscillation frequency, demonstrating positive frequency dependence. At any given frequency, the G′ value increased initially and then decreased with elevating HQP concentration, peaking at a 12% addition level. This improvement could be linked to HQP-mediated myofibrillar protein aggregation, which facilitated the establishment of a compact and stable gel microstructure, thereby augmenting the viscoelastic attributes of the chicken breast batters (Jiang et al., 2025). However, at a 15% addition level, excess HQP competed with myofibrillar proteins for free water, diluting the continuous phase and reducing batter viscosity. Consequently, structural stability was compromised, ultimately leading to a decline in G′ value (Lv et al., 2021; Petracci, Mudalal, Babini, & Cavani, 2014).

### 3.5 Low-field NMR

**Fig. 2:**
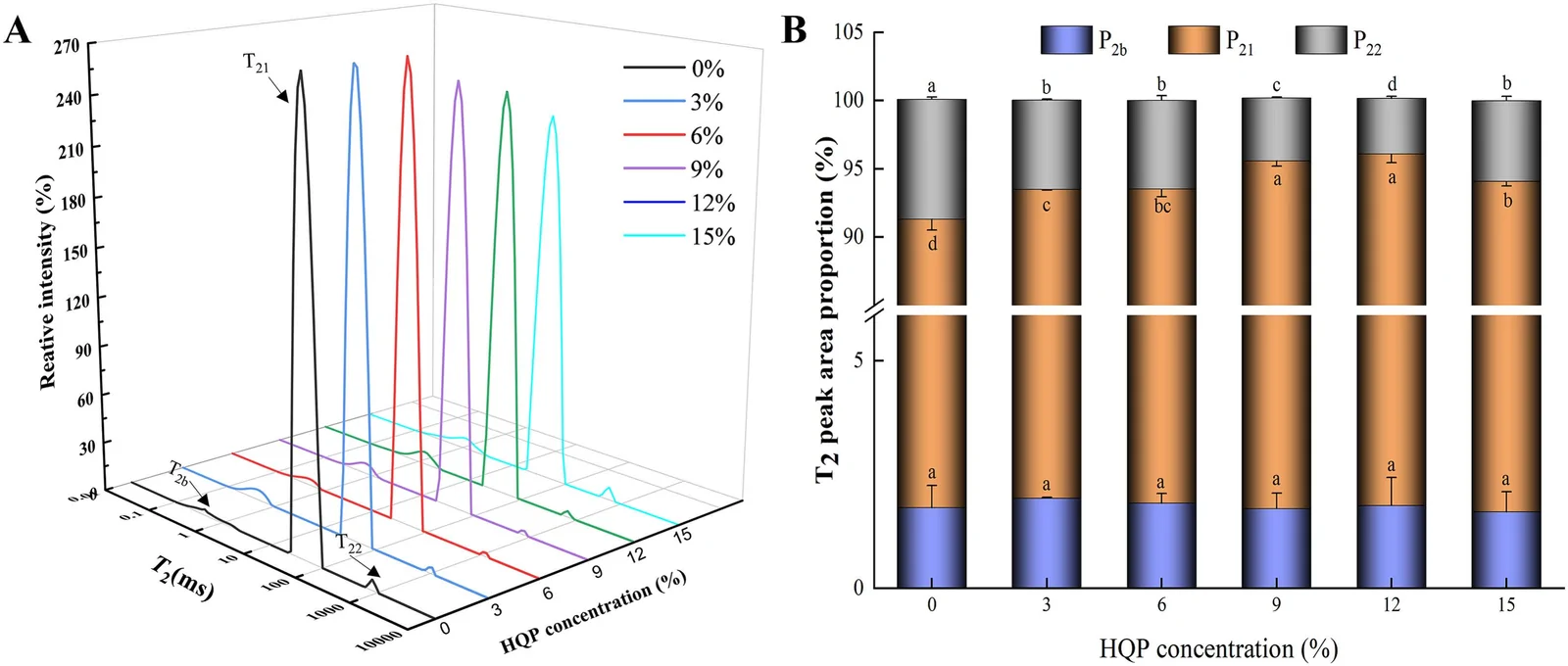
Effects of HQP on the T_2_ relaxation times (A), and T_2_ relaxation peak area proportion (B) of the chicken breast batter gel. Note: Different letters indicate significant differences among groups (*P* < 0.05).

Water distribution and migration in meat products can be rapidly and nondestructively characterized by LF-NMR technology. LF-NMR transverse relaxation time (T₂) measurements enable the identification of three distinct water populations in meat batters: T₂b (0–10 ms, bound water), T₂₁ (10–100 ms, immobilized water), and T₂₂ (400–800 ms, free water) (Zhao et al., 2024). As illustrated in Fig. 2A, the addition of HQP at varying concentrations significantly altered the T₂ relaxation spectra of chicken breast batter gels, indicating that HQP markedly influenced water distribution and migration within the gel matrix. The T₂ relaxation time decreased significantly as the HQP concentration increased from 3% to 12%, reaching its minimum at the 12% addition level (Table 3). This observation can be largely explained by the improved WHC of HPH-treated quinoa protein (Zhao et al., 2023). Xie et al. (2022) described analogous findings, demonstrating that the addition of ultra-high pressure modified soybean protein significantly reduced the T₂ relaxation time of minced pork gels. Conversely, at a 15% addition level, the T₂ relaxation time increased, possibly because excessive HQP tended to aggregate, thereby disrupting the regularly arranged gel network structure and compromising water retention (Jiang et al., 2025). Therefore, moderate addition of HQP could effectively reduce water mobility and enhance water retention, a trend in line with the cooking yield observations (Fig. 1A).

**Table 3:** Effect of HQP on T_2_ relaxation time of the chicken breast batter gel.

HQP concentration (%)	T_2_ relaxation/ms		
	T_2b_	T_21_	T_22_
0	1.82 ± 0.12^a^	43.73 ± 1.47^a^	733.67 ± 11.21^a^
3	1.62 ± 0.11^a^	41.54 ± 2.01^a^	655.48 ± 20.08^b^
6	1.33 ± 0.06^b^	35.26 ± 1.24^b^	591.87 ± 15.89^c^
9	1.25 ± 0.12^bc^	31.74 ± 0.54^c^	573.63 ± 26.51^c^
12	0.84 ± 0.08^d^	28.74 ± 0.31^d^	498.67 ± 19.32^d^
15	1.12 ± 0.06^c^	30.52 ± 1.06^c^	536.41 ± 31.18^cd^

Note: Different letters in the same column indicate a significant difference (*P* < 0.05).

The relative proportions of the three water populations (Fig. 2B) further revealed that with progressive elevation of HQP levels from 3% to 12%, the percentage of immobilized water (P₂₁) rose significantly from 89.55% to 94.28% (*P* < 0.05), accompanied by a concomitant decrease in free water (P₂₂), indicating that HQP incorporation fundamentally transformed the water mobility landscape within the gel structure. Mechanistically, the elevated P₂₁ fraction likely reflects the dual role of HQP as both a water-binding agent and a network densifier: the hydrophilic moieties exposed during HPH treatment increase the thermodynamic affinity for water, while the HQP-myofibrillar protein interactions physically entrap water molecules within smaller, more tortuous interstitial compartments, thereby restricting their rotational and translational mobility (Cen et al., 2022). The inverse relationship between P₂₂ and cooking yield (Fig. 1A) further supports the interpretation that HQP suppresses water exudation by reducing the fraction of water susceptible to thermal syneresis. This is consistent with established NMR principles, wherein P₂₁ serves as a proxy for water entrapped within the gel network and correlates positively with water-holding capacity, whereas P₂₂ reflects water loosely held within larger pores and prone to migration under thermal or mechanical stress. Previous studies have established that P₂b exerts negligible influence on physicochemical properties and water-holding capacity, and that P₂₁ is significantly positively correlated with water retention, whereas P₂₂ exhibits a negative correlation (Zhao et al., 2022). Taken together, these findings indicate that the improved cooking yield (Fig. 1A) arises not merely from the intrinsic water-binding capacity of quinoa protein, but from HQP-induced structural reorganization of the gel network that restricts water mobility through geometric confinement.

### 3.6 FT-IR

**Fig. 3:**
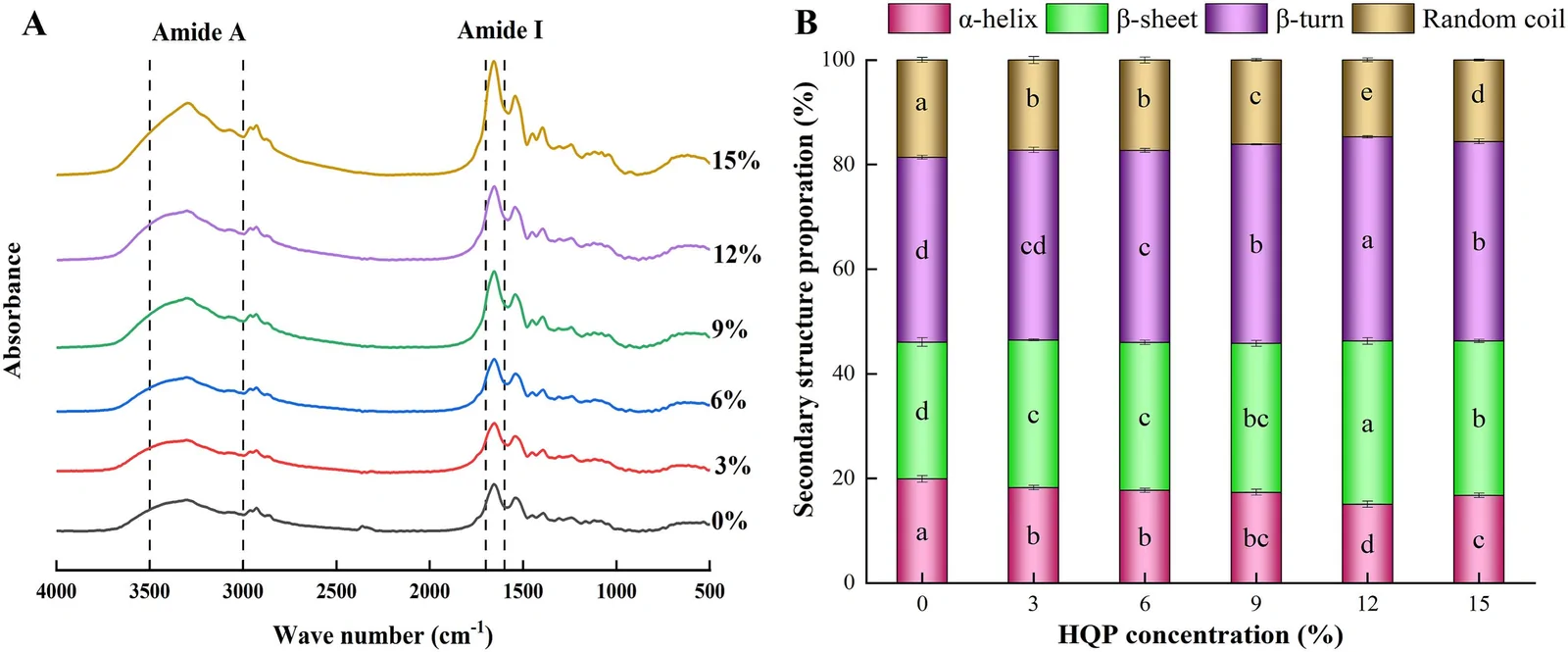
Effects of HQP on the FTIR spectroscopy (A), and protein secondary structure proportion (B) of the chicken breast batter gel. Note: Different letters indicate significant differences among groups (*P* < 0.05).

FT-IR spectroscopy (400–4000 cm^−1^) represents a fast, non-destructive analytical technique widely employed to assess variations in protein secondary structure. The Amide A band (N—H or O—H stretching vibrations, 3000–3500 cm^−1^) provides valuable insights into protein–water interactions, with higher peak wavenumbers indicative of weaker interactions (Liu, Wang, et al., 2023; Liu, Yang, et al., 2023). As documented in Fig. 3A, HQP addition remarkably altered the Amide A absorption maximum of chicken breast batter gels. As the HQP concentration increased, the Amide A peak shifted to lower wavenumbers, indicating enhanced protein–water interactions. This could stem from the presence of HQP, which facilitates the construction of abundant and intensified hydrogen bonds with water molecules (Wang et al., 2024). This observation aligns with the concurrent enhancement of gel strength (Fig. 1B) reported herein, indicating that HQP incorporation promotes hydrogen bond formation in chicken batter gels.

As a distinctive fingerprint absorption band for protein secondary structure, the Amide *I* band enables quantitative quantification of the proportional distribution of various protein secondary structural elements. Typically, the region of 1650–1660 cm^−1^ is assigned to α-helix; 1610–1640 cm^−1^ and 1670–1680 cm^−1^ are ascribed to β-sheet, 1660–1670 cm^−1^ and 1680–1700 cm^−1^ are assigned to β-turn, and 1640–1650 cm^−1^ is associated with random coil (Zhao et al., 2022). The structural integrity of protein gels is inherently tied to the compositional heterogeneity exhibited by these secondary structures. As shown in Fig. 3B, increasing the HQP concentration from 3% to 12% was associated with a progressive decline in α-helix and random coil fractions, along with a concomitant significant enhancement of β-sheet and β-turn contents. The observed structural transition is attributable to HQP-induced exposure of hydrophobic groups in myofibrillar proteins, which enhanced intermolecular hydrophobic interactions, facilitated protein unfolding, and destabilized α-helix structures (Cen et al., 2022). Additionally, the elevated β-sheet percentage contributed to improvements in water-holding capacity and gel strength primarily because β-sheets constitute a highly ordered lamellar structure stabilized by extensive inter- and intramolecular hydrogen bonds. This ordered architecture serve as a critical driving force for protein crosslinking and network densification during gelation. The lamellar arrangement promotes tight molecular packing, thereby facilitating the formation of a uniform, dense, and continuous 3D gel network. Moreover, the hydrogen-bonding potential inherent to β-sheet structures not only reinforces the mechanical stability of the protein network but also facilitates water immobilization through interactions with water molecules, thereby converting free water into bound water within the gel matrix (Wang et al., 2021; Yang et al., 2017). This further supports the notion that the increased cooking yield (Fig. 1A) and gel strength (Fig. 1B) reported herein stem from β-sheet-mediated protein interactions that generate tighter crosslinks. Ma et al. (2025) documented analogous findings, demonstrating that HPH modified chickpea protein promoted the conformational transition from α-helix to β-sheet in chicken batter proteins, thereby facilitating the formation of a tightly packed and firm gel network. Collectively, these results demonstrate that HQP effectively induced conformational transitions in myofibrillar proteins, promoted α-helix unfolding while enhancing intermolecular aggregation, thereby facilitating the formation of a dense and robust chicken breast batter gel network.

### 3.7 Microstructure

**Fig. 4:**
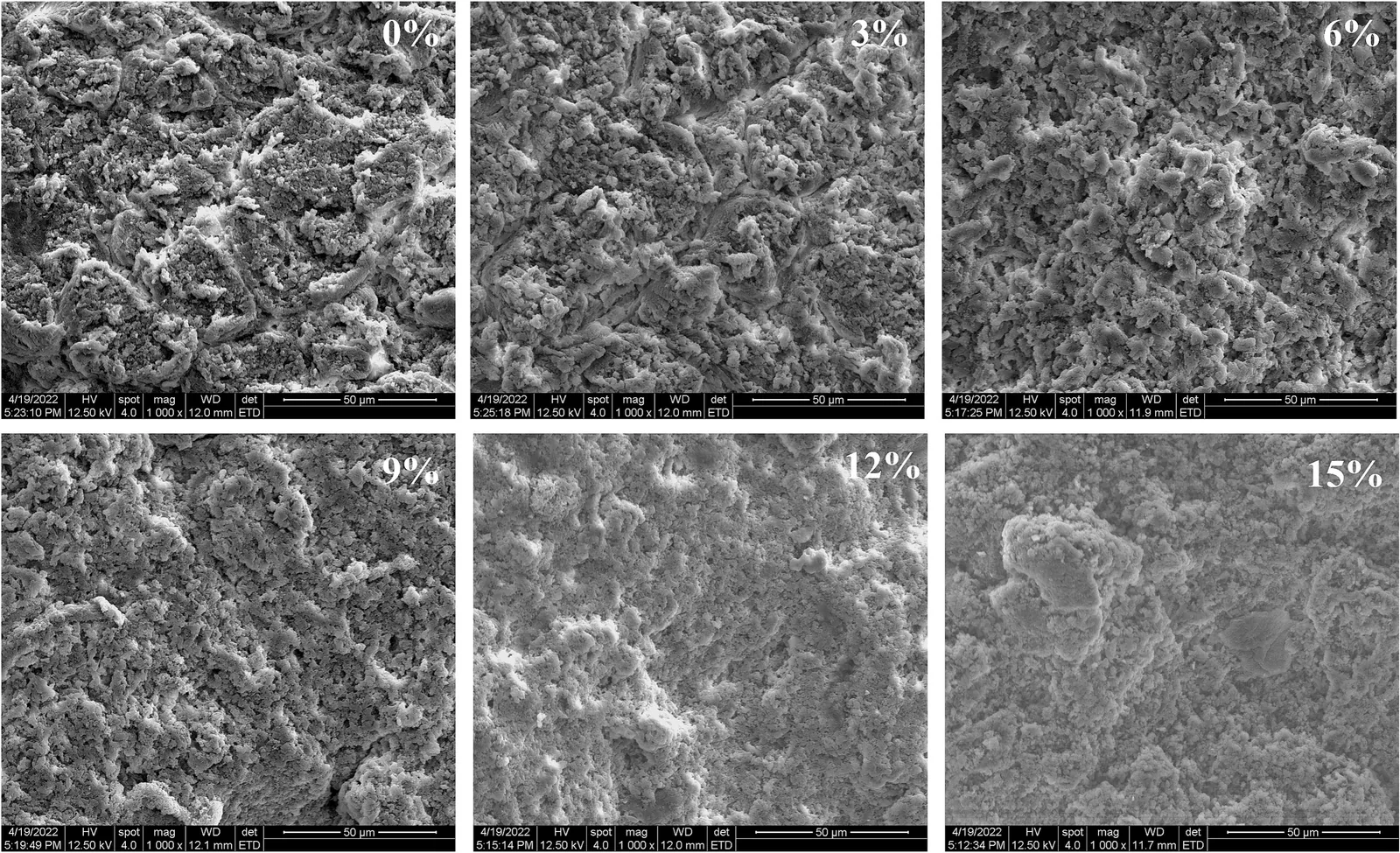
Effects of HQP on the microstructure of the chicken breast batter gel. Note: Different letters indicate significant differences among groups (*P* < 0.05).

As shown in Fig. 4, the control exhibited a coarse, loose gel microstructure characterized by large, irregular voids and fragmented protein aggregates—structural features consistent with its inferior mechanical strength and elevated free water fraction (Fig. 2B). These macroscopic voids likely functioned as stress-concentration loci and channels for water migration, explaining the poor water retention and low fracture resistance of the control gel. With increasing HQP concentration, the microstructure progressively became denser, more homogeneous, and highly ordered. At the optimal addition level of 12%, the gel network appeared remarkably compact, well-defined, and smooth. This pronounced improvement is predominantly attributed to the molten globule state adopted by quinoa protein upon HPH modification, whereby partial chain unfolding of quinoa protein effectively converts large, irregular pores into smaller, more uniformly distributed interstitial compartments, leading to the development of a closely packed, isotropic gel microstructure in conjunction with myofibrillar proteins (Colmenero, 2000). Moreover, the enhanced solubility and reduced particle size of quinoa protein induced by HPH treatment further enabled intimate spatial integration within the myofibrillar scaffold, minimizing phase separation and promoting isotropic gelation, thereby fostering the establishment of a stable, uniform gel network (Lin et al., 2019). Consistent with these observations, Dong et al. (2023) confirmed that ultrasound-modified pea protein induced the emergence of a more homogeneous and compact gel matrix in pork myofibrillar protein gels. This microstructural transition provides the morphological basis for the concurrent physicochemical improvements. The reduced void volume directly mirrors the LF-NMR-observed water redistribution (Fig. 2B)—specifically, the decrease in free water (P₂₂) and increase in immobilized water (P₂₁)—as the denser network geometrically confines water within smaller, more tortuous pores. Concurrently, elimination of large structural defects explains the pronounced gains in gel strength and chewiness at 12% HQP, since the homogeneous, fine-stranded network enables uniform stress distribution, underpinning the 70.8% enhancement in gel strength and the disproportionate improvement in chewiness (Johansson et al., 2024).

## 4 Conclusions

HQP addition effectively ameliorated the gel characteristics of chicken breast batters by altering the secondary structure, water mobility, rheological attributes, and microstructure. Specifically, HQP addition drove the α-helix to β-sheet structural transition, yielding a microstructure with fine, evenly dispersed pores, thereby improving textural attributes, storage modulus (G′), and gel strength. Furthermore, the resulting homogeneous and highly cross-linked gel network effectively reduced water mobility, increased the proportion of immobilized water, and enhanced cooking yield. Collectively, these findings indicate that HQP represents a promising functional ingredient for improving the gel quality of chicken breast batter products, with considerable potential for the production of premium minced chicken products.

## CRediT authorship contribution statement

**Yanyan Zhao:** Writing – original draft, Funding acquisition. **Xinyuan Yao:** Software, Methodology. **Qizhao Han:** Methodology. **Zhaohui Wei:** Supervision, Resources. **Ligong Zhai:** Investigation. **Shengming Zhao:** Writing – review & editing, Conceptualization. **Guoyuan Xiong:** Project administration, Funding acquisition.

## Declaration of competing interest

The authors declare that they have no known competing financial interests or personal relationships that could have appeared to influence the work reported in this paper.

## Data Availability

Data will be made available on request.

## References

[bb0005] Ampofo J., Ngadi M. (2022). Ultrasound-assisted processing: Science, technology and challenges for the plant-based protein industry. Ultrasonics Sonochemistry.

[bb0010] Cao Y., Xia T., Zhou G., Xu X. (2012). The mechanism of high pressure-induced gels of rabbit myosin. Innovative Food Science & Emerging Technologies.

[bb0015] Cen K., Yu X., Gao C., Yang Y., Tang X., Feng X. (2022). Effects of quinoa protein Pickering emulsion on the properties, structure and intermolecular interactions of myofibrillar protein gel. Food Chemistry.

[bb0020] Colmenero F.J. (2000). Relevant factors in strategies for fat reduction in meat products. Trends in Food Science & Technology.

[bb0025] Dakhili S., Abdolalizadeh L., Hosseini S.M., Shojaee-Aliabadi S., Mirmoghtadaie L. (2019). Quinoa protein: Composition, structure and functional properties. Food Chemistry.

[bb0030] Dong C., Zhao J., Wang L., Jiang J. (2023). Modified pea protein coupled with transglutaminase reduces phosphate usage in low salt myofibrillar gel. Food Hydrocolloids.

[bb0035] Ekezie F.G.C., Cheng J.H., Sun D.W. (2018). Effects of nonthermal food processing technologies on food allergens: A review of recent research advances. Trends in Food Science & Technology.

[bb0040] Ge Q., Wu Y., Yuan N., Jia Z., Liu R., Lu F., Kang Z. (2023). Effect of high pressure homogenization-modified soy 11S globulin on the gel and rheological properties of pork myofibrillar protein. Foods.

[bb0045] Ghafouri-Oskuei H., Javadi A., Saeidi-Asl M.R., Azadmard-Damirchi S., Armin M., Riazi F., Savadkoohi S. (2022). Mechanical attributes, colloidal interactions, and microstructure of meat batter influenced by flaxseed flour and tomato powder. Meat Science.

[bb0050] Gul O., Akgun A., Maribao I.P., Parlak M.E., Saricaoglu F.T., Simsek S. (2025). Mechanism for improving acid-induced hazelnut protein gels through high-pressure homogenization: Effect on structural, rheological and gelling properties. Foods.

[bb0055] Hou G., Han Y., Zhou F., Li Y., Zou Z., Zhang L., Zheng B. (2025). Enhancing pork meat batter with curdlan gum-guar gum composite microgels: Impact of fat replacer on gel properties, water distribution, and sensory perception. Food Chemistry.

[bb0060] Jiang T., Zhao Y., Huang M., Zhang Z., Mao Y., Zuo H. (2025). Influence of interactions between drawing soy protein and myofibrillar proteins on gel properties. Foods.

[bb0065] Johansson M., Karlsson J., van den Berg F.W., Ström A., Ahrne L., Sandström C., Langton M. (2024). Effect of cellulose-rich fibres on faba bean protein gels is determined by the gel microstructure. Food Hydrocolloids.

[bb0070] Kang Z.L., Bai R., Lu F., Zhang T., Gao Z.S., Zhao S.M., Ma H.J. (2022). Effects of high pressure homogenization on the solubility, foaming, and gel properties of soy 11S globulin. Food Hydrocolloids.

[bb0075] Lee H.C., Jang H.S., Kang I., Chin K.B. (2017). Effect of red bean protein isolate and salt levels on pork myofibrillar protein gels mediated by microbial transglutaminase. LWT-Food Science and Technology.

[bb0080] Li J., Chen Y., Dong X., Li K., Wang Y., Wang Y., Bai Y. (2021). Effect of chickpea (*Cicer arietinum L.*) protein isolate on the heat-induced gelation properties of pork myofibrillar protein. Journal of the Science of Food and Agriculture.

[bb0085] Lin D., Zhang L., Li R., Zheng B., Rea M., Miao S. (2019). Effect of plant protein mixtures on the microstructure and rheological properties of myofibrillar protein gel derived from red sea bream (*Pagrosomus major*). Food Hydrocolloids.

[bb0090] Liu Q., Geng R., Zhao J., Chen Q., Kong B. (2015). Structural and gel textural properties of soy protein isolate when subjected to extreme acid pH-shifting and mild heating processes. Journal of Agricultural and Food Chemistry.

[bb0095] Liu S., Wang Z., Zheng J., Sun W., Xiao Z., Shao J.H. (2023). Effects of direct current magnetic field co-treated with stirring on gel properties of chicken batter: Hydration and textural properties. Journal of Food Engineering.

[bb0100] Liu Y., Yang L., Zhao S., Zhao Y., Kang Z., Zhu M., Ma H. (2023). Effect of Artemisia sphaerocephala krasch gum on the functional properties of pork batters. Journal of Texture Studies.

[bb0105] Lv Y., Xu L., Su Y., Chang C., Gu L., Yang Y., Li J. (2021). Effect of soybean protein isolate and egg white mixture on gelation of chicken myofibrillar proteins under salt/−free conditions. LWT-Food Science and Technology.

[bb0110] Ma Y., Sang X., Wang C., Zhang J., Guo X. (2025). Effects of high-pressure homogenization modified chickpea protein addition on the gel properties of minced chicken. LWT-Food Science and Technology.

[bb0115] Niu H., Xia X., Wang C., Kong B., Liu Q. (2018). Thermal stability and gel quality of myofibrillar protein as affected by soy protein isolates subjected to an acidic pH and mild heating. Food Chemistry.

[bb0120] Petcharat T., Benjakul S. (2018). Effect of gellan incorporation on gel properties of bigeye snapper surimi. Food Hydrocolloids.

[bb0125] Petracci M., Mudalal S., Babini E., Cavani C. (2014). Effect of white striping on chemical composition and nutritional value of chicken breast meat. Italian Journal of Animal Science.

[bb0130] Wang K.N., Sun H., Cui Z.H., Wang J.H., Hou J.Y., Lu F.P., Liu Y.H. (2024). Synergistic effects of microbial transglutaminase and apple pectin on the gelation properties of pea protein isolate and its application to probiotic encapsulation. Food Chemistry.

[bb0135] Wang S., Miao S., Sun D.W. (2024). Modifying structural and techno-functional properties of quinoa proteins through extraction techniques and modification methods. Trends in Food Science & Technology.

[bb0140] Wang X., Wang L., Yang K., Wu D., Ma J., Wang S., Sun W. (2021). Radio frequency heating improves water retention of pork myofibrillar protein gel: An analysis from water distribution and structure. Food Chemistry.

[bb0145] Wang X., Xia M., Zhou Y., Wang L., Feng X., Yang K., Sun W. (2020). Gel properties of myofibrillar proteins heated at different heating rates under a low-frequency magnetic field. Food Chemistry.

[bb0150] Wang Y., Wang J.L., Li K.E., Yuan J.J., Chen B., Wang Y.T., Bai Y.H. (2025). Effect of chickpea protein modified with combined heating and high-pressure homogenization on enhancing the gelation of reduced phosphate myofibrillar protein. Food Chemistry.

[bb0155] Xie J.J., Zou X.L., Li Y.P., Kang Z.L., Ma H.J. (2022). Effects of high-pressure-modified soy 11S globulin on the gel properties and water-holding capacity of pork batter. International Journal of Food Science and Technology.

[bb0160] Yang Q.L., Lou X.W., Wang Y., Pan D.D., Sun Y.Y., Cao J.X. (2017). Effect of pH on the interaction of volatile compounds with the myofibrillar proteins of duck meat. Poultry Science.

[bb0165] Yang Z., de Campo L., Gilbert E.P., Knott R., Cheng L., Storer B., Hemar Y. (2022). Effect of NaCl and CaCl_2_ concentration on the rheological and structural characteristics of thermally-induced quinoa protein gels. Food Hydrocolloids.

[bb0170] Zhang X.H., Pan H., Jiang X., Shi W.Z. (2023). Study on the mechanism of soy protein isolate to improve quality of reduced-salt surimi gel: Focus on gel quality, protein structure, and in vitro digestibility. Food Chemistry: X.

[bb0175] Zhang Y., Zhang J., Chen Q., He N., Wang Q. (2022). High-moisture extrusion of mixed proteins from soy and surimi: Effect of protein gelling properties on the product quality. Foods.

[bb0180] Zhao S., Liu Y., Yuan X., Zhao Y., Kang Z., Zhu M., Ma H. (2022). Effect of low-frequency alternating magnetic field on the rheological properties, water distribution and microstructure of low-salt pork batters. LWT-Food Science and Technology.

[bb0185] Zhao S., Wang Z., Zhai L., Zhao Y., Huang M., Zheng H., Xiong G. (2025). Modification of reduced-sodium carp myofibrillar protein by a combined strategy with ultrasound and MgCl_2_ treatment: Physicochemical, structural and functional properties. Ultrasonics Sonochemistry.

[bb0190] Zhao S., Yang L., Chen X., Zhao Y., Ma H., Wang H., Su A. (2024). Modulation of the conformation, water distribution, and rheological properties of low-salt porcine myofibrillar protein gel influenced by modified quinoa protein. Food Chemistry.

[bb0195] Zhao Y., Yuan Y., Yuan X., Zhao S., Kang Z., Zhu M., Ma H. (2023). Physicochemical, conformational and functional changes of quinoa protein affected by high-pressure homogenization. LWT-Food Science and Technology.

[bb0200] Zheng H.B., Xu B.C., Xu X.L., Li C., Bolumar T., Zhen Z.Y. (2021). Gelation of chicken batters during heating under high pressure. Innovative Food Science & Emerging Technologies.

